# Hospitalization for urinary tract infections in Japan, 2010–2015: a retrospective study using a national inpatient database

**DOI:** 10.1186/s12879-021-06735-y

**Published:** 2021-10-09

**Authors:** Akahito Sako, Hideo Yasunaga, Hiroki Matsui, Kiyohide Fushimi, Hidekatsu Yanai, Yoshiaki Gu, Norio Ohmagari

**Affiliations:** 1 Department of Internal Medicine, Kohnodai Hospital, National Center for Global Health and Medicine, 1-7-1 Kohnodai, Ichikawa, Chiba 272-8516 Japan; 2 Department of Clinical Epidemiology and Health Economics, School of Public Health, Graduate School of Medicine, The University of Tokyo, 7-3-1 Hongo, Bunkyo-ku, Tokyo, Japan; 3 Department of Health Informatics and Policy, Tokyo Medical and Dental University Graduate School of Medicine, 1-5-45 Yushima, Bunkyo-ku, Tokyo, Japan; 4 Department of Infectious Diseases, Tokyo Medical and Dental University Graduate School of Medical and Dental Sciences, 1-5-45 Yushima, Bunkyo-ku, Tokyo, Japan; 5 Disease Control and Prevention Center, Center Hospital, National Center for Global Health and Medicine, 1-21-1 Toyama, Shinjuku-ku, Tokyo, Japan

**Keywords:** Urinary tract infection, Pyelonephritis, Incidence, Mortality, Nationwide

## Abstract

**Background:**

Urinary tract infections (UTI) are common and can have severe consequences. However, there are few recent large-scale studies about them. We aimed to determine the incidence of hospitalization for UTI and to elucidate patient characteristics, clinical practice, and clinical outcomes by drawing on a Japanese nationwide database.

**Methods:**

This was a retrospective observational study using a national database that covers half the acute care inpatients in Japan. Patients aged ≥ 15 years who were hospitalized for UTI were eligible. We did not include patients with lower UTI such as cystitis. We investigated the annual number of patients hospitalized in Japan, those patients’ characteristics, and risk factors for in-hospital mortality.

**Results:**

We identified 232,396 eligible patients from 31 million records of discharge between April 2010 and March 2015. The average age was 73.5 years and 64.9% of patients were female. The estimated annual number of hospitalizations because of UTI was 106,508. The incidence was 6.8 per 10,000 for men and 12.4 for women. The median medical care cost was 4250 USD. In-hospital mortality was 4.5%. Risk factors of poor survival included male sex, older age, lower bed capacity, non-academic hospital, admission in winter, higher Charlson Comorbidity Index score, low body mass index, coma on admission, ambulance use, disseminated intravascular coagulation, sepsis, renal failure, heart failure, cerebrovascular diseases, pneumonia, malignancies, use of anti-diabetic drugs, and use of corticosteroid or immunosuppressive drugs.

**Conclusions:**

We found that older patients of both sexes accounted for a significant proportion of those hospitalized for UTI. The clinical and economic burden of UTI is considerable.

**Supplementary Information:**

The online version contains supplementary material available at 10.1186/s12879-021-06735-y.

## Background

Urinary tract infections (UTI) such as acute pyelonephritis are the second most common type of infection requiring hospitalization after lower respiratory tract infections [1]. Previous studies have shown that the complications and mortality of acute pyelonephritis are associated with significant healthcare burdens [2]. The incidence of hospitalization for acute pyelonephritis in the USA was 11.7 per 10,000 for women and 2.4 for men in 1997 [3]. The direct and indirect costs of acute pyelonephritis were estimated as 2.14 billion USD in 2000 [2].

UTIs may progress to severe conditions such as sepsis, shock, and disseminated intravascular coagulation (DIC). The mortality of patients hospitalized for UTI or acute pyelonephritis is reportedly 1–9% [2, 4–7]. When accompanied by bacteremia, the mortality of acute pyelonephritis is 10–20% [8]. Risk factors for death are older age, immunosuppression, bedridden status, septic shock, DIC, disturbance of consciousness, and recent use of antibiotics [5, 8]. Early identification of patients with acute pyelonephritis at high risk of death may contribute to more effective treatment [8].

Nationwide studies that investigate the prevalence, patient characteristics, and clinical course of UTI are lacking. We therefore conducted a large-scale retrospective study using an administrative and clinical inpatient database to determine the incidence, patient characteristics, and clinical outcomes of hospitalization for UTI in Japanese acute care hospitals.

## Methods

### Diagnosis procedure combination (DPC) database

The participating hospitals voluntarily submit discharge abstract and administrative claims data to the DPC Research Group for clinical epidemiology research [9–12]. The validity of diagnoses recorded in the DPC database is generally high: the sensitivity and specificity of primary diagnoses have been reported to be 78.9% and 93.2%, respectively [13]. As of 2012, the number of participating hospitals was 1098, and the total number of beds 388,000, accounting for 43% of all beds in acute care hospitals in Japan. In 2012, the number of hospital admissions in the database was 6.85 million, representing approximately 50% of all admissions from Japanese acute care hospitals.

### Patient selection and variables

The DPC data contain a maximum of 12 diagnoses per patient and are listed according to International Classification of Diseases 10th Revision (ICD-10) codes and text data. They consist of four main diagnoses, four comorbidities on admission, and four complications during hospitalization. We retrospectively examined data on patients who had UTI-related ICD-10 codes (Additional file 1: Table S1) in the diagnoses for admission from the fiscal years 2010 to 2014. We also included patients who had DIC or sepsis related ICD-10 codes in the diagnosis for admission and had UTI in the comorbidities on admission because DIC and sepsis are common comorbidities of UTI, and reimbursements are higher for DIC and sepsis than for UTI. Although we did not review all diagnoses in the Japanese text data, we confirmed the diagnoses using text data if necessary. We did not include hospitalization for lower UTI such as cystitis, prostatitis, or urethritis because these conditions differ substantially from pyelonephritis or upper UTI. Additionally, we did not include nosocomial UTIs because we could not determine the date on which UTI developed after admission or the association of UTI and catheter indwelling. We excluded patients aged under 15 years and those who did not receive antibiotics on the day of admission. Thus, only patients aged ≥ 15 years hospitalized for UTI alone or UTI with DIC or sepsis were eligible.

We used the following data: age; sex; body weight and height; discharge status; academic hospital or community hospital; number of beds; underlying diseases (Additional file 1: Table S1); pregnancy status; procedures and surgeries; medication use during hospitalization; medical costs. We divided body mass index (BMI) into the following five groups: underweight, BMI < 18.50 kg/m^2^; low–normal weight, 18.50–22.99; high–normal weight, 23.00–24.99; overweight, 25.00–29.99; and obese, ≥ 30.00. Consciousness levels on admission and discharge were expressed as Japan Coma Scale (JCS) grades. We categorized these into four groups [14]: grade 0, alert; grade 1, drowsy, but awake without any stimuli; grade 2, somnolent, but arousable with stimulation; and grade 3, coma. We calculated the Charlson Comorbidity Index (CCI) scores as the burden of comorbidities [15, 16].

### Statistical analysis

We estimated the number of hospitalizations for UTI (Y) in Japan by the following equation:

$$\text{Y}=\sum _{i=1}^{k}\frac{XiNi}{ni}$$

where *N* is the number of beds in all acute care hospitals in Japan, *n* is the number of beds in DPC hospitals, and *X* is the observed number of patients with UTI in DPC hospitals. We stratified hospitals by every 100 beds to adjust for the fact that DPC hospitals are skewed toward larger hospitals. We calculated the annual incidence of UTI by dividing the estimated number of patients with UTI (Y) by Japanese population in 2012.

For univariate analysis, we used Student’s *t*-test, the χ^2^ test, or Fisher’s exact test as appropriate. We performed multivariable logistic regression analysis to identify the risk factors associated with in-hospital mortality. In the multivariable regression model, the independent variables included age, sex, and variables that were clinically relevant and significantly associated with in-hospital death by univariate analyses. We fitted multivariable logistic regression analyses for in-hospital mortality with generalized estimating equations to account for within-hospital clustering [17]. We calculated variance inflation factors for independent variables to avoid multicollinearity between the independent variables. We considered *P* < 0.05 to denote statistical significance. All statistical analyses were performed by IBM SPSS Statistical package Version 25 (IBM, Armonk, NY, USA).

## Results

### Patient characteristics and incidence of hospitalization

**Fig. 1:**
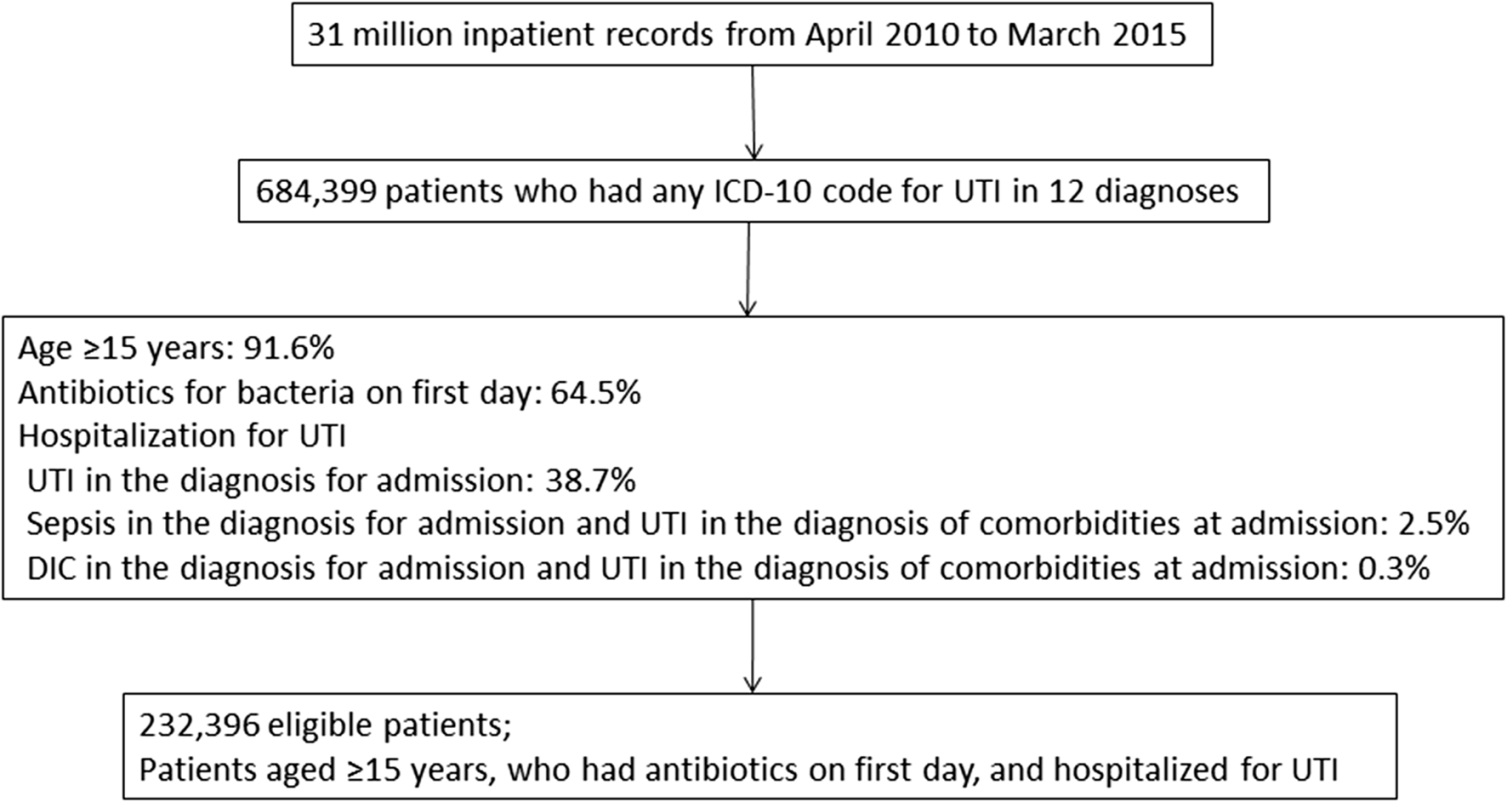
Study flow diagram showing selection of patients hospitalized for UTI

Among 31 million discharges from April 2010 to March 2015, 684,339 patients had any ICD-10 code for UTI among their 12 diagnoses. Finally, 232,396 patients were eligible (Fig. 1).

The patients’ characteristics are shown in Table 1. Mean age was 73.4 years (Standard Deviation: 17.4). There were more female than male patients in all age groups. Young age (15–39 years) accounted for only 3.0% of male but 8.8% of female patients. Overall, 1% of female patients in all age categories were pregnant and 11.1% of female patients aged 15–39 years. The average age of pregnant patients was 28.0 years and average week of pregnancy 23.4. The mean BMI was 21.9 kg/m^2^ (SD 4.3) and average CCI score 0.9 (SD 1.3).

**Table.1:** Patient characteristics (*n* = 232,396)

	*N*	(%)
Sex		
Female	150,780	64.9
Pregnant	1510	0.6
Age (years)		
15–19	2068	0.9
20–29	6569	2.8
30–39	7034	3.0
40–49	9355	4.0
50–59	14,345	6.2
60–69	29,572	12.7
70–79	56,997	24.5
80–89	77,409	33.3
90–99	277,89	12.0
≥ 100	1258	0.5
Academic hospital	22,831	9.8
Ambulance use	72,946	31.4
Hospital bed capacity		
20–199	27,796	12.0
200–299	33,370	14.4
300–399	54,321	23.4
400–499	38,716	16.7
500–599	33,301	14.3
600–699	19,634	8.4
≥ 700	23,512	10.1
Missing	1746	0.8
Body mass index		
< 18.5	41,631	17.9
18.5–22.9	85,933	37.0
23.0–24.9	30,271	13.0
25.0–29.9	32,023	13.8
≥ 30	8405	3.6
Missing	34,133	14.7
Charlson Comorbidity Index		
0	141,142	60.7
1	22,954	9.9
2	49,882	21.5
3	7616	3.3
≥ 4	10,802	4.6
Japan Coma Scale grade on admission		
Grade 0 (alert)	177,491	76.4
Grade 1 (drowsy)	39,135	16.8
Grade 2 (somnolent)	11,452	4.9
Grade 3 (coma)	4312	1.9
Underlying diseases		
Cerebrovascular disease	26,457	11.4
Dementia	20,655	8.9
Pneumonia	18,475	7.9
Sepsis	41,711	17.9
Diabetes mellitus	45,820	19.7
Ischemic heart disease	14,857	6.4
Disseminated intravascular coagulation	8814	3.8
Heart failure	18,736	8.1
Renal failure	18,101	7.8
Chronic respiratory disease	10,115	4.4
Chronic liver disease	5624	2.4
Schizophrenia	4980	2.1
Neuromuscular dysfunction of bladder	13,012	5.6
Hyperplasia of prostate	14,276	6.1
Urolithiasis	24,758	10.7
Malignancies	28,682	12.3
Urological malignancies	12,828	5.5
Obstructive and reflux uropathy	28,624	12.3
Therapeutic interventions		
Mechanical ventilation	3644	1.6
Transurethral stenting	19,177	8.3
Percutaneous nephrostomy	3781	1.6
ESWL (Extracorporeal shock wave lithotripsy)	2782	1.2
Hemodialysis	2881	1.2
Intensive care unit admission	3578	1.5

Patients aged ≥ 65 years more frequently had underlying diseases related to UTI such as diabetes (21.1% vs. 15.0%), neurogenic bladder (5.9% vs. 4.4%), and prostate hyperplasia (19.2% vs. 9.9%, male only) than younger patients. They also more frequently had complications of UTI such as sepsis (19.4 vs. 13.1%) and DIC (4.1% vs. 2.6%).

Annual numbers of hospitalizations for UTI were 8.8–10.4 per 100 beds in hospitals with < 500 beds and 5.0–7.6 in those with ≥ 500 beds.

**Fig. 2:**
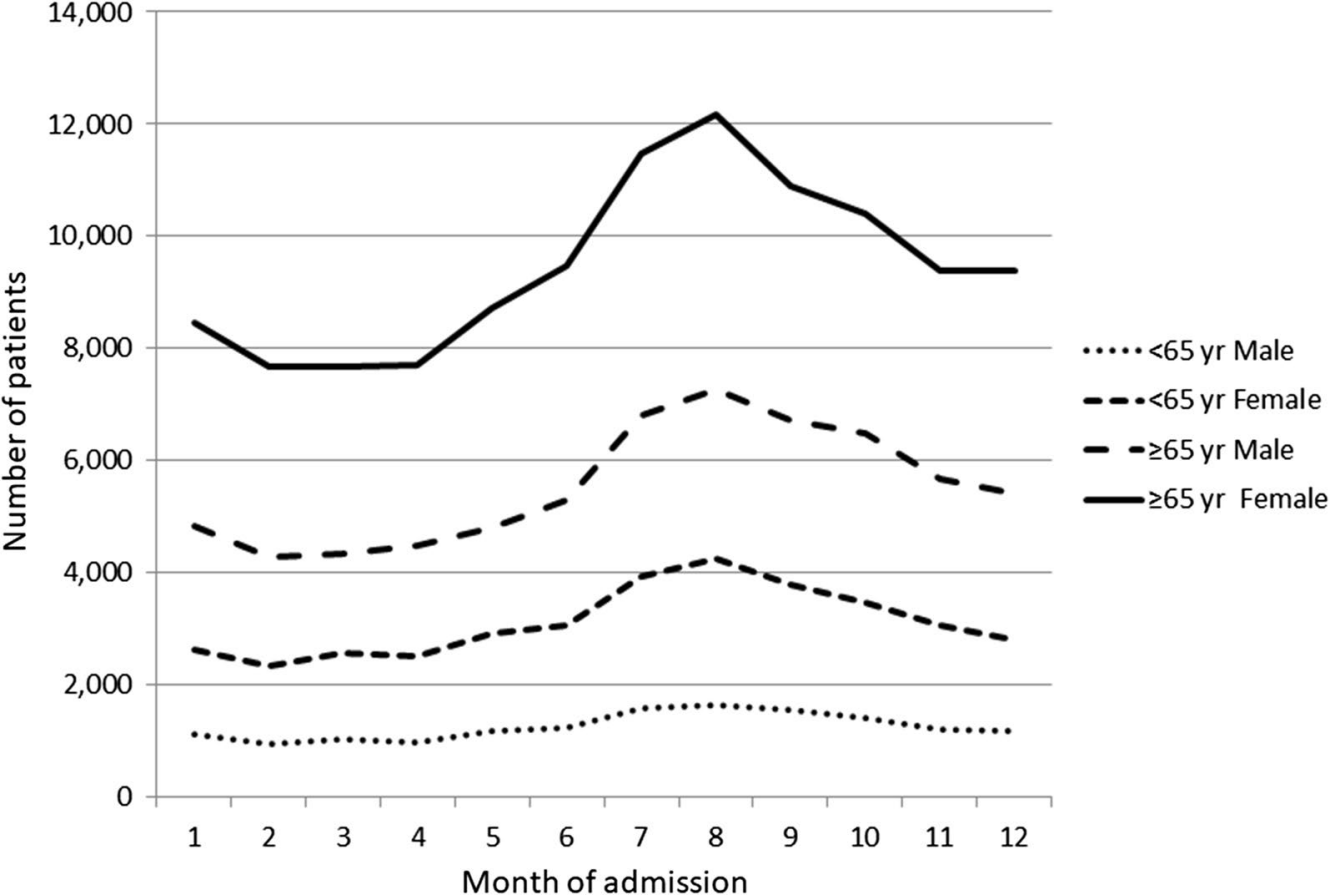
Seasonal changes in number of hospitalizations for UTI stratified by age and sex

We observed clear seasonal changes every year (Fig. 2). UTIs were more prevalent (29.4%) in summer (from June to August) followed by 27.6% in the fall, 22.0% in winter, and 21.1% in spring. We observed this seasonality in both sexes and in patients of all ages, even when categorized in 10-year intervals.

**Fig. 3:**
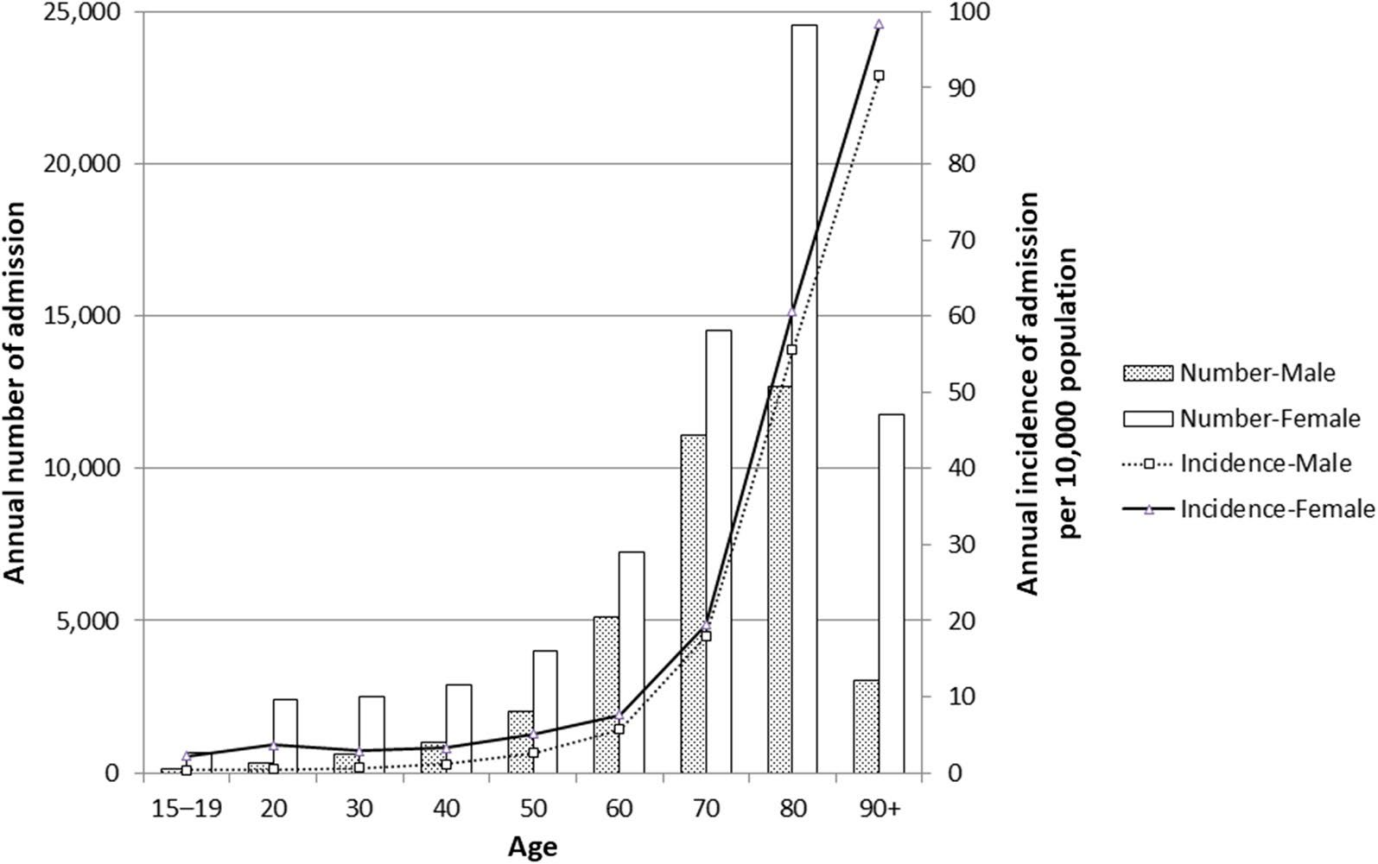
Estimated annual number and incidence of admissions for UTI in Japan in 2012 stratified by sex

In 2012, the estimated numbers of hospitalizations for UTI in Japan were 35,969 for men and 70,539 for women. The estimated annual incidence of hospitalization for UTI was 6.8 per 10,000 for men and 12.4 per 10,000 for women. Incidences in male teens and men in their 50 s were 0.4–2.6 per 10,000, respectively, and those in female teens and women in their 50 s were 2.3–5.1 per 10,000, respectively. Incidences in patients over 60 were similar between men (5.7–91.6) and women (7.6–98.5) and much higher than younger patients (Fig. 3).

### Clinical courses and risk factors for in-hospital mortality

Median length of hospitalization was 12 days (interquartile range: 11 days). Median medical cost was 4250 USD (interquartile range: 3960 USD; 1 USD = 100 JPY, during the study period, 1USD = 80–120 JPY). Median length of stay and medical cost in patients < 65 and ≥ 65 years were 8 days and 13 days, respectively, and 3154 USD and 4630 USD, respectively.

Among the patients with obstructive and reflux uropathy, 34.2% had urolithiasis, 8.4% had urological malignancies, 6.3% had neuromuscular dysfunction of the bladder, and 5.7% had hyperplasia of the prostate. Among the patients with urolithiasis, 43.2% had transurethral stenting, 8.7% had extracorporeal shock wave lithotripsy, and 5.6% had percutaneous nephrostomy. Among the patients with urological malignancies, 6.3% had transurethral stenting and 4.8% had percutaneous nephrostomy.

Antibiotics on admission and during hospitalization are shown in Table 2. Single antibiotics were given to 93.3% of study patients on the first day. As for anti-DIC drugs during hospitalization, unfractionated heparin was given to 4.9% of study patients, thrombomodulin-alfa to 2.1%, and antithrombin III to 1.1%. Any anti-DIC drug, including heparin, was given to 8.3% (4.6% excluding heparin). As to vasopressors during hospitalization, norepinephrine, dopamine, and dobutamine were given to 2.8%, 4.8%, and 0.6% of study patients, respectively. Blood cultures were performed on 50.9% on admission.

**Table.2:** Antibiotics on admission and during hospitalization (*n* = 232,396)

	1st day (%)	During hospitalization (%)
Penicillins	21.6	30.7
First generation cephalosporins	5.1	11.5
Second generation cephalosporins	18.5	24.0
Third generation cephalosporins	37.9	51.5
Fourth generation cephalosporins	4.4	6.1
Carbapenems	10.7	18.7
Category of broadness of beta-lactams		
Carbapenems	10.7	18.7
Antipseudomonal beta-lactams	23.8	30.2
Third generation cephalosporins without antipseudomonal activity	31.8	45.0
Penicillins with beta-lactamase inhibitor	7.9	11.5
Other beta-lactams	24.1	37.2
Anti-MRSA drugs	0.9	3.3
Aminoglycosides	1.6	3.3
Fluoroquinolones	4.5	30.2

*MRSA* methicillin-resistant *Staphylococcus aureus*

The overall in-hospital mortality was 4.5%. Crude in-hospital mortality of patients aged < 65 and ≥ 65 years were 1.0% and 5.6%. Results of multivariable logistic regression analysis for in-hospital mortality are shown in Table 3. The factors associated with poor survival were male sex, older age, lower bed capacity, non-academic hospital, admission in winter, higher CCI, low BMI, coma on admission, ambulance use, DIC, sepsis, renal failure, heart failure, cerebrovascular diseases, pneumonia, malignancies, anti-diabetic drugs including insulin, and corticosteroid or immunosuppressive drugs.

**Table.3:** Results of logistic regression analysis for in-hospital mortality

	Crude mortality (%)	Adjusted odds ratio	95% confidence interval			*P*
Sex						
Female	4.3	0.88	0.83	–	0.93	< 0.001
Male	5.0	1.00	Ref.			
Age (years)						
≥ 90	9.8	16.51	10.85	–	25.14	< 0.001
80–89	6.2	10.20	6.72	–	15.50	< 0.001
70–79	3.5	6.32	4.16	–	9.59	< 0.001
60–69	2.2	4.47	2.93	–	6.82	< 0.001
50–59	1.3	3.77	2.44	–	5.83	< 0.001
40–49	0.6	2.47	1.50	–	4.06	< 0.001
≤ 39	0.2	1.00	Ref.			
Hospital bed capacity						
≥ 700	3.6	0.78	0.65	–	0.94	0.008
600–699	4.3	0.79	0.66	–	0.94	0.008
500–599	4.3	0.79	0.68	–	0.92	0.002
400–499	4.4	0.82	0.71	–	0.95	0.010
300–399	4.6	0.90	0.79	–	1.03	0.123
200–299	5.2	1.01	0.87	–	1.16	0.947
≤ 199	5.1	1.00	Ref.			
Type of hospital						
Academic	3.0	0.73	0.64	–	0.84	< 0.001
Community	4.7	1.00	Ref.			
Season of admission						
Winter	5.3	1.11	1.04	–	1.20	0.004
Autumn	4.4	0.99	0.92	–	1.06	0.705
Summer	4.0	0.89	0.83	–	0.96	0.002
Spring	4.6	1.00	Ref.			
Charlson Comorbidity Index						
≥ 4	12.6	1.83	1.62	–	2.06	< 0.001
3	7.6	0.89	0.78	–	1.02	0.096
2	6.2	1.06	0.98	–	1.16	0.161
1	3.9	0.94	0.85	–	1.03	0.184
0	3.2	1.00	Ref.			
Body mass index (kg/m^2^)						
≥ 30.0	1.7	0.75	0.63	–	0.88	0.001
25.0–29.9	2.1	0.71	0.65	–	0.78	< 0.001
23.0–24.9	2.5	0.74	0.68	–	0.81	< 0.001
18.5–22.9	3.7	1.00	Ref.			
< 18.5	7.5	1.67	1.58	–	1.78	< 0.001
Japan Coma Scale grade on admission						
Grade 3 (coma)	26.3	4.51	4.00	–	5.09	< 0.001
Grade 2 (somnolent)	13.4	2.35	2.13	–	2.59	< 0.001
Grade 1 (drowsy)	6.8	1.46	1.35	–	1.57	< 0.001
Grade 0 (alert)	2.9	1.00	Ref.			
Ambulance use						
Yes	6.8	1.12	1.05	–	1.18	< 0.001
No	3.5	1.00	Ref.			
Disseminated intravascular coagulation						
Yes	20.4	2.92	2.66	–	3.20	< 0.001
No	3.9	1.00	Ref.			
Sepsis						
Yes	11.3	2.35	2.19	–	2.53	< 0.001
No	3.1	1.00	Ref.			
Renal failure						
Yes	10.6	2.29	2.12	–	2.47	< 0.001
No	4.0	1.00	Ref.			
Heart failure						
Yes	11.3	1.88	1.71	–	2.06	< 0.001
No	3.9	1.00	Ref.			
Cerebrovascular disease						
Yes	6.5	1.20	1.11	–	1.29	< 0.001
No	4.3	1.00	Ref.			
Pneumonia						
Yes	16.4	3.35	3.13	–	3.60	< 0.001
No	3.5	1.00	Ref.			
Malignancies						
Yes	8.8	3.17	2.89	–	3.48	< 0.001
No	3.9	1.00	Ref.			
Use of anti-diabetic drugs						
Yes	6.7	1.47	1.38	–	1.56	< 0.001
No	4.1	1.00	Ref.			
Use of corticosteroid or immunosuppressive drugs						
Yes	11.6	2.73	2.56	–	2.92	< 0.001
No	3.8	1.00	Ref.			

All of the above variables were used for calculation of the adjusted odds ratios

## Discussion

This nationwide retrospective study involving 232,396 hospitalizations to acute care hospitals in Japan for UTI investigated both epidemiological and clinical data. Mean age was 73.5 years. Estimated number of hospitalizations for UTI in 2012 was 106,508. In-hospital mortality was 4.5%, and we identified several risk factors for death.

There have been several nationwide studies on hospitalization for acute pyelonephritis [3, 18]. However, to the best of our knowledge, no such studies have been published since 2000. Additionally, our study includes more clinical information, such as patient characteristics, treatments, and outcomes.

A South Korean nationwide study reported an annual incidence of hospitalization for acute pyelonephritis in 1997–1999 of 1.2 per 10,000 population for men and 10.0 for women [18]. A population-based study in the USA from 1997 to 2001 reported annual rates of female outpatients and inpatients with acute pyelonephritis of 12–13 and 3–4 per 10,000 population, respectively, and 2–3 and 1–2 per 10,000 among male outpatients and inpatients, respectively [19]. We estimated annual incidences of hospitalization for UTI in Japan as 6.8 per 10,000 population for men and 12.4 for women. Although young women are at high risk of UTI, most patients hospitalized for UTI were older women and men, which would be partly attributable to the increasing aging in Japan. In our study, patients aged ≥ 65 years more frequently had underlying diseases related to their UTIs.

Previous studies have shown that pregnancy is associated with a higher incidence of acute pyelonephritis or UTI and that these conditions increase the risk of preterm birth [20–23]. In 2006, 29,000 pregnant women were hospitalized in the USA for acute pyelonephritis [22]. Pregnant patients comprised from 18 to 31% of hospitalized patients with acute pyelonephritis and aged < 40 years in Canada in 1990 [20]. In the current study, 11% of female patients aged 15–39 years were pregnant.

Diabetes is also a risk factor for acute pyelonephritis [20]. One-fifth of the patients in our study had diabetes, which is compatible with the previous study. Sodium-glucose co-transporter 2 (SGLT-2) inhibitors can increase the risk of UTI because of excretion of glucose into the urine [24]. Because SGLT-2 inhibitors were just introduced in Japan in 2014, the incidence of UTI in diabetic patients will increase as prescriptions for SGLT-2 inhibitors increase.

Several studies have shown that the incidence of acute pyelonephritis is highest in summer [18, 19, 22]. However, a study in the USA showed that both sexes are more likely to be hospitalized for acute pyelonephritis in winter and that crude mortality in male patients is highest in winter and spring [21]. We found seasonal variations in incidence and a peak in summer in both sexes and all age categories. Although the reasons for seasonal fluctuations are unknown, possibilities include changes in behavior, environmental, and microbial factors [18]. In our study, winter was an independent risk factor for death. A previous USA study showed death from all natural causes increased in winter [25], which supports our results.

A Korean nationwide study in 1997-99 showed an average duration of hospitalization for acute pyelonephritis of 7.9 days. The total annual medical cost for it accounted for 0.2% of national medical costs [18]. Nearly 2 billion USD for community-acquired and nosocomial UTIs in the USA is a significant health economic burden [26]. We found that mean medical cost of hospitalization for UTIs was 6,230 USD and the estimated annual cost in Japan 663 million USD. Taking into account the medical cost of nosocomial UTIs and outpatient care, and indirect costs such as absenteeism, the economic burden of UTIs is significant. Aging has a major impact on disease burden because of the associated multiple comorbidities. We showed patients aged ≥ 65 years more frequently had severe complications of UTI, were hospitalized for longer, and accumulated greater cost.

Mortality varies between studies because of differences in clinical settings, definitions of UTI, comorbidities, and severity. In a retrospective study of acute pyelonephritis in Greece showed septic shock occurred in 20% of patients and the mortality was 13.3% [8]. In a Japanese multi-center study of obstructive pyelonephritis caused by urolithiasis, 12% of the patients had DIC, 15% needed vasopressors, 67% underwent urinary drainage, and the overall mortality was 2.3% [5]. Prospective observational study of complicated pyelonephritis in Spain showed the mortality was 6.5% [6]. Mortality in our study was 4.5%. Our patients were not limited to complicated UTI nor tertiary care hospitals; thus, rates of septic shock and DIC were not as high as those in other studies.

We identified the additional risk factors of male sex, low BMI, and use of anti-diabetic drugs to those identified in previous studies. Several studies have shown that low BMI and being underweight are risk factors for death from pneumonia [27, 28] and another study showed lower mortality in obese patients with pneumonia [29]. Few studies have examined the association between BMI and mortality from UTI and none have identified a significant association [29]. Our study is the first to show a significant impact of underweight on mortality from UTI.

This study has several limitations. First, the DPC database does not contain some clinical information, including vital signs and laboratory and imaging data. We could not confirm the diagnosis of UTI based on symptoms, pyuria, and positive urine culture. We could not confirm obstruction of the urinary tract using imaging data from CT or ultrasonography. Although pyelonephritis and tubulointerstitial nephritis had the same ICD-10 codes, we were able to exclude tubulointerstitial nephritis using data on antibiotic use on admission and diagnoses in the Japanese text data. Because we selected eligible patients on the basis of diagnoses on admission, we may have overlooked patients with UTI and severe concurrent diseases other than DIC or sepsis on admission, such as renal failure and disturbance of consciousness, thus possibly underestimating the numbers of hospitalizations for UTI. Second, we intended to investigate patients with upper UTI or pyelonephritis; however, many patients had unspecified UTI diagnoses. We think most of these patients had upper UTI or pyelonephritis because it is common to use the diagnosis of UTI for pyelonephritis in Japan, and patients with cystitis rarely need hospitalization. Third, the DPC database is limited to acute care hospitals, and participating hospitals are skewed toward large academic hospitals. To adjust for this, we stratified hospitals by bed capacity. Fourth, the DPC database only contains inpatient information and is not linked with other databases such as outpatient records and vital statistics. Thus, we were unable to investigate antibiotics and urinary catheter before admission and survival after discharge, limiting our findings to short-term clinical course and in-hospital mortality. Fifth, because length of hospital stay of acute care in Japan was more than twice as long as other developed countries [30], it is difficult to compare length of hospitalization with studies in other countries. Despite these limitations, in this nationwide study we have determined the estimated annual incidence, patient characteristics, clinical practice, and in-hospital mortality of hospitalizations for UTI in Japan based on recent clinical and epidemiological data.

## Conclusions

Although young women are well known to be at relatively high risk of UTI, we found that older patients of both sexes account for a significant proportion of those hospitalized for UTI. The clinical and economic burden of UTI is considerable. In-hospital mortality is relatively low; however, clinician should carefully manage comorbidities and risk factors.

## Supplementary Information

**Additional file 1: Table S1.** ICD-10 codes for UTI and underlying diseases.

## Data Availability

The datasets used and/or analysed during the current study are not publicly available for ethical reasons as the data are patient data. The datasets are available from the corresponding author on reasonable request, pending ethical approval.

## Abbreviations

UTI: Urinary tract infections
DIC: Disseminated intravascular coagulation
DPC: Diagnosis procedure combination
ICD-10: International Classification of Diseases 10th Revision
BMI: Body mass index
JCS: Japan Coma Scale
CCI: Charlson Comorbidity Index
SD: Standard deviation
SGLT-2: Sodium-glucose co-transporter 2
MRSA: Methicillin-resistant *Staphylococcus aureus*
ESWL: Extracorporeal shock wave lithotripsy
