# Supplementary material for: Hospitalization for urinary tract infections in Japan, 2010–2015: a retrospective study using a national inpatient database
Source: BMC Infect Dis. 2021 Oct 9;21:1048. doi: 10.1186/s12879-021-06735-y (PMC8502312; doi:10.1186/s12879-021-06735-y)
Supplement: Supplementary file 1 — Additional file 1: Table S1. ICD-10 codes for UTI and underlying diseases. [file 12879_2021_6735_MOESM1_ESM.docx]

**Supplementary Table.** ICD-10 codes for UTI and underlying diseases

| Disease category | ICD-10 code |
| --- | --- |
| UTI-related diagnoses |  |
| Acute tubulo-interstitial nephritis including pyelonephritis | N10.x |
| Chronic tubulo-interstitial nephritis including pyelonephritis | N11.x |
| Tubulo-interstitial nephritis including pyelonephritis,  not specified as acute or chronic | N12.x |
| Pyonephrosis | N13.6 |
| Renal and perinephric abscess | N15.1 |
| Urinary calculus including calculous pyelonephritis | N20.9 |
| Urinary tract infection, site not specified | N39.0 |
| Underlying diseases |  |
| Cerebrovascular diseases | I60.x–64.x, I69.x |
| Dementia | F00.x–03.x, F05.1, G30.x, G31.0, G31.1 |
| Pneumonia | J12.x–18.x, J69.0 |
| Sepsis | A02.1, A40.x, A41.x |
| Diabetes mellitus | E10.x–14.x |
| Ischemic heart diseases | I20.x–25.x |
| Disseminated intravascular coagulation | D65 |
| Heart failure | I11.0, I13.0, I13.2, I50.x |
| Renal failure | I12.0, I13.1, I13.2, N17.x, N18.x, N19.x |
| Chronic respiratory diseases | J40.x–47.x, J60.x–67.x |
| Chronic liver diseases | B18.x, I85.x, I86.4, I98.2, K70.2–70.4, K71.1, K71.3–71.5, K71.7, K72.x–74.x, K76.6, K76.7 |
| Schizophrenia | F20.x-29.x |
| Neuromuscular dysfunction of bladder, not elsewhere classified | N31.x |
| Hyperplasia of prostate | N40 |
| Urolithiasis | N20.x-23.x |
| Malignancies | Cxx.x |
